# Economic burden of varicella in children 1–12 Years of age in Hungary, 2011–2015

**DOI:** 10.1186/s12879-017-2575-6

**Published:** 2017-07-14

**Authors:** Z. Meszner, Z. Molnar, E. Rampakakis, H. K. Yang, B. J. Kuter, Lara J. Wolfson

**Affiliations:** 1 St. László Hospital for Infectious Diseases, National Institute of Child Health, Budapest, Hungary; 2 National Center for Epidemiology, Budapest, Hungary; 3 JSS Medical Research, Montreal, QC Canada; 4 Merck & Co, Inc., Center for Observational and Real-World Evidence (CORE), MAILSTOP WP97-A243, 770 Sumneytown Pike, West Point, PA 19486 USA

## Abstract

**Background:**

Although live-attenuated varicella-zoster virus (VZV) vaccines have been proven to be safe and effective in preventing varicella and real-word evidence shows routine childhood immunization programs are effective in dramatically reducing varicella associated morbidity and mortality, varicella vaccine is not included in the National Immunization Program (NIP) in Hungary. The purpose of this study was to evaluate the clinical and economic burden associated with varicella in Hungary.

**Methods:**

This was a multicenter, retrospective, chart review study of patients aged 1–12 years with a primary varicella diagnosis between 2011 and 2015. Healthcare resource utilization (HCRU) associated with varicella, unit costs, and work loss were used to estimate direct and indirect costs. All costs are presented in 2015 HUF / Euros (€).

**Results:**

156 children with varicella were included (75 outpatients, 81 inpatients), with a mean age of 4.4 (SD: 2.0) and 3.7 (SD: 2.1) years, respectively. One or more complications were reported by 12.0% of outpatients and 92.6% of inpatients, the most common being dehydration, skin and soft tissue infections, pneumonia, keratoconjunctivitis, and cerebellitis. HCRU estimates included use of over-the-counter (OTC) medications (96.0% outpatients, 53.1% inpatients), prescription medications (9.3% outpatients, 70.4% inpatients), tests/procedures (4.0% outpatients, 97.5% inpatients), and consultation with allied health professionals (2.7% outpatients, 30.9% inpatients). The average duration of hospital stay (inpatients) was 3.6 (95% CI: 3.2, 4.1) days. The total combined direct and indirect cost per varicella case was 228,146.7 Hungarian Forint (HUF)/€ 736.0 for inpatients and 49,790.6 HUF/€ 106.6 for outpatients. The overall annual cost of varicella in Hungary for children aged <15 years in 2015 was estimated at 1,903,332,524.3 HUF/ € 6,139,980.4.

**Conclusion:**

Varicella is associated with substantial clinical burden in Hungary, resulting in the utilization of a significant amount of healthcare resources. These results support the need for routine vaccination of all healthy children to reduce the varicella-associated disease burden.

## Background

Varicella, also known as chickenpox, is caused by varicella-zoster virus (VZV) and is one of the most infectious diseases occurring in childhood. Symptoms of the viral infection, including fever, malaise, headache, and abdominal pain, do not typically present before a 10- to 21-day incubation period [1, 2], which ultimately results in a generalized pruritic vesicular rash. In addition, varicella infection may occasionally lead to complications, some of the most common being neurologic complications such as cerebellitis and encephalitis, skin and soft tissue complications, gastrointestinal or lower respiratory involvement, and pneumonia [3–5].

The annual worldwide incidence of varicella is estimated between 2 and 16 cases per 1000 persons [6–8], where regional variations are commonly observed due to factors such as age, immunosuppression and climate. In Hungary, the incidence of varicella was reported by the Hungarian National Center for Epidemiology (NCE) to be 3.96 cases per 1000 persons in 2010, for a total of 39,602 cases [9], and reported a similar number of varicella cases (*n* = 40,853) for 2015 [10]. Both numbers are most likely an underestimate of the true number of varicella cases given that many mild cases are not reported or seen by medical personnel. Although no data exist on the age-specific seroprevalence of varicella antibodies in Hungary, data from bordering Slovakia indicates that more than 96% of the population has been exposed by age 20 [11], and 100% by age 40 [12], which suggests that, in the absence of a vaccination program, the annual number of cases is expected to be (on average) similar to the size of the Hungarian birth cohort, currently 91,700 [13]. This estimate is also supported by sero-epidemiology datasets from 12 other European countries indicating that the annual number of varicella cases is approximately equal to the birth cohort, suggesting that the number of varicella cases in Hungary may be underreported by a factor of 2 [14].

In Hungary, treatment guidelines for immunocompetent patients presenting with varicella are limited and include suggestions of cool showering, increased fluid intake, use of talcum powder, and cutting nails short [15]. Hospitalization is indicated for complicated cases including, but not limited to, those associated with severe skin lesions, toxic state, abdominal/chest pain, neurological complaints, atypical rashes, and continuing fever. Antiviral treatment with acyclovir (ACV) is not recommended as a prophylactic, but suggested for treating complications and for shortening the duration of symptoms.

Varicella vaccines, most of which contain a live-attenuated virus of the Oka strain, have existed since the mid-1980s, and offer protection for at least 14 years [16–21]. Varicella vaccine is well tolerated and effective, and is licensed in various countries for use in healthy children, typically over the age of 12 months. Several countries have included varicella vaccine as part of their immunization programs, and as a result, these countries have observed a dramatic decline in morbidity and mortality associated with varicella. As an example, since the introduction of varicella vaccines in their national immunization programs, the US and Canada have observed declines of 88% and 81–88%, respectively, in hospitalizations associated with varicella [22, 23]. In Hungary, although recommended by NCE the varicella vaccine is optional for children and not covered or reimbursed by the Ministry of Human Capacities [24, 25],

The primary objective of this study was to describe the burden of illness associated with varicella in Hungary, including morbidity, healthcare resource utilization (HCRU), and the associated cost among children 1–12 years of age, diagnosed with varicella, who sought either outpatient or inpatient care between 2011 and 2015. The results of this study aim to provide policy makers in Hungary with local evidence of the healthcare use and costs associated with varicella that could be offset through a national varicella vaccination plan. This study also provides critical data required to populate health economic models and cost-effective analyses of interventions for varicella in Hungary.

## Methods

### Study design

This was a multicenter, observational study that evaluated the burden of illness associated with varicella through the use of a retrospective chart review design and was conducted in accordance with the generally accepted standards of Good Pharmacoepidemiology Practice (GPP). In line with the local regulations, the study was approved by the Hungarian Ministry of Health (MOH) and no review by Independent Ethics Committees (IECs) was required. Patient consent was also not required, as data were retrospectively collected in an anonymous manner by treating physicians and identified only by an encrypted patient number.

### Case selection

Based on the recommendations of the Hungarian primary care pediatricians’ society and the board of the society of infectious diseases, 15 potential physician sites were selected to participate in the study. Those that expressed preliminary interest (*n* = 8) were surveyed to estimate the number of patients potentially eligible for the study within the five years prior to study initiation and, eventually, 6 sites (5 public hospitals - Miskolci Semmelweis Hospital and University Teaching Hospital [MISEK], Markusovszky University Hospital, Pándy Kálmán Hospital, Szent György University Teaching Hospital, St. László Hospital for Infectious Diseases - and 1 private practice of a general practitioner) agreed to participate in the study and contributed patient charts. Of these, 5 were in urban areas [Budapest (*n* = 2), Miskolc, Székesfehérvár, Szombathely] and 1 was in a rural area (Gyula). For case selection, investigators were instructed to screen patient charts in their practices for eligibility for the study starting from the most recent year and going back as much as five years. The date of first primary varicella infection was defined as the index date, and each patient’s chart was reviewed from this date until the resolution of the disease occurred or the last date of contact, if the resolution date was unavailable. This observational study aimed to include the charts of 150 patients as a sample of convenience, equally divided between outpatient and inpatient settings.

### Study population

Patients 1–12 years of age with a primary varicella diagnosis between 2011 and 2015, in roughly equal numbers of outpatients and inpatients, were targeted for inclusion. The outpatient group included patients who visited either the doctor’s office (family doctor, general practitioner, pediatrician, and infectious disease specialist), outpatient clinic/department of hospital, or emergency department (ER) without hospitalization for varicella, and inpatients were defined as those admitted to a hospital for their primary varicella. Patients who had received prior varicella vaccination and who had a diagnosis of breakthrough varicella, or a second case of varicella, were excluded from the study.

### Outcome measures

The varicella-related clinical complications that were evaluated in this study included, but were not limited to, skin and soft tissue infection, meningitis, encephalitis, pneumonia, sepsis, acute osteomyelitis, septic arthritis, cerebellitis, keratoconjunctivitis, hepatitis, nephritis, febrile seizure, dehydration, severe pain, and coagulation disorder. The distribution of these complications was assessed descriptively by calculating the number and proportion of patients with at least one complication. Any other complications aside from those listed above were coded using the Medical Dictionary for Regulatory Activities (MedDRA), version 18.0, and were reported by preferred term. HCRU was assessed using the number and proportion of patients using each resource, the frequency of use, and the duration of healthcare resource use for varicella and varicella-related complications. The healthcare resources that were evaluated involved outpatient visits, allied healthcare contacts, doctor’s visits, tests/procedures performed, prescription medications prescribed, over-the-counter (OTC) medications, hospitalizations, ER visits/stays, and intensive care unit (ICU) stays. The direct cost of HCRU was determined by multiplying the amount of resources used per patient by the unit cost of each resource, as assessed by local expert opinion (Table 1). The indirect costs were calculated as the loss of revenue of caregivers who cared for varicella infected children, using the national average income statistics reported by the Organization for Economic Co-Operation (OECD) [26] and the number of work days missed by the caregiver. The number of work days that were missed was estimated as the total days spent in the hospital/ICU for inpatients during this study and 2.5 days for outpatients, as estimated in previous studies [27, 28]. All costs are presented in 2015 HUF / Euros (€) [29].

**Table 1:** Key unit costs (HUF / €) for healthcare resources

	Outpatients (*N* = 75)	
	Mean Cost	
Healthcare resource		
Visits to doctor’s office	HUF €	1000–5,000^a^ 3–16
Visits to ER	HUF €	15,000–30,000^a^ 48–97
Visits to hospital outpatient clinic	HUF €	10,000 32
Day of hospitalization	HUF €	20,000–40,000^a^ 64–129
Day of ICU stay	HUF €	50,000–100,000^a^ 161–322

*ER* emergency room, *HUF* Hungarian Forint, *ICU* intensive care unit

^a^For cost analyses, the mean value was used

### Statistical methods

All enrolled patients were included in the statistical analysis, and subgroup analysis was performed for outpatients and inpatients. Descriptive statistics were produced to address all study objectives, which included measures of central tendency (mean) and dispersion statistics (SD and 95% CI) for continuous variables, and frequency distributions (number and percentage) for categorical variables. Due to the low number of cases in some outcome measures, logarithmic transformation was used for the calculation of 95% CIs. All statistical analyses were performed using SAS® software version 9.4 (SAS Institute Inc., Cary, NC, USA).

## Results

A total of 156 eligible patients were enrolled in this study, of whom 75 (48.1%) were outpatients and 81 (51.9%) were inpatients.

Table 2 reports the cohort’s demographics and disease characteristics at varicella diagnosis. The mean age was 4.4 (SD: 2.0) and 3.7 (SD: 2.1) years for outpatients and inpatients, respectively, with almost equal distribution by gender. Most outpatients (68.0%) had <50 skin lesions compared with 2.5% of inpatients, while the majority of inpatients (72.8%) presented with 50 to 249 skin lesions compared with 21.3% of outpatients. A total of 12.0% of outpatients and 92.6% of inpatients experienced at least one varicella-associated complication. Of the patients experiencing complications, 22.2% of outpatients experienced more than one complication in contrast to 41.3% of inpatients. Of note, no patient was considered immunocompromised in the outpatient group, whereas 4.9% of inpatients had at least one immunocompromising condition.

**Table 2:** Patient and disease characteristics at varicella diagnosis

	Outpatients (*N* = 75)	Inpatients (*N* = 81)
Patient characteristics		
Age, years, mean (SD)	4.4 (2.0)	3.7 (2.1)
Gender, n (%)		
Male	33 (44.0%)	45 (55.6%)
Female	42 (56.0%)	36 (44.4%)
Area of residence, n (%)		
Urban	71 (94.7%)	53 (65.4%)
Rural	4 (5.3%)	28 (34.6%)
BMI, kg/m^2^, mean (SD)	15.3 (2.0)	15.8 (2.6)
Calendar year of diagnosis, n (%)		
2011	2 (2.7%)	4 (4.9%)
2012	13 (17.3%)	18 (22.2%)
2013	17 (22.7%)	9 (11.1%)
2014	43 (57.3%)	40 (49.4%)
2015	0 (0.0%)	10 (12.3%)
Disease characteristics		
Maximum number of skin lesions during rash, n (%)		
< 50	51 (68.0%)	2 (2.5%)
50–249	16 (21.3%)	59 (72.8%)
250–500	7 (9.3%)	20 (24.7%)
> 500	1 (1.3%)	0 (0.0%)
Patients with at least one complication, n (%)	9 (12.0%)	75 (92.6%)
Number of complications, n (% among those experiencing complications)		
1 complication	7 (77.8%)	44 (58.7%)
> 1 complication	2 (22.2%)	31 (41.3%)
Patients who were immunocompromised^a^, n (%)	0 (0.0%)	4 (4.9%)

*BMI* body mass index, *SD* standard deviation

^a^Patients were considered immunocompromised if they had at least one of the following conditions: HIV/AIDS, congenital immunodeficiency, received system steroids, or had any other immunocompromised condition listed in their medical history

**Fig. 1:**
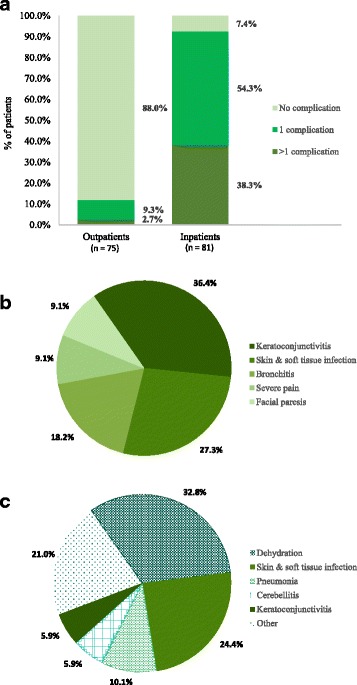
Types of complications associated with varicella. **a** Percentage of patients with complications*. **b** Most common complications - Outpatients^†^. **c** Most common complications - Inpatients^†‡^

Figure 1 presents the types of complications associated with varicella that were observed in outpatient and inpatient groups. The 5 most common complications for outpatients were keratoconjunctivitis (36.4% of all complications), skin and soft tissue infection (27.3%), bronchitis (18.2%), severe pain (9.1%) and facial paresis (9.1%), whereas for inpatients, complications included dehydration (32.8%), skin and soft tissue infection (24.4%), pneumonia (10.1%), cerebellitis (5.9%) and keratoconjunctivitis (5.9%). The inpatient group reported the following additional complications: otitis media (2.5%), sepsis (1.7%), febrile seizure (1.7%), severe pain (1.7%), coagulation disorder (1.7%), enteritis (1.7%), scarlet fever (1.7%), encephalitis (0.8%), bronchitis (0.8%), epididymitis (0.8%), epilepsy (0.8%), gastroenteritis (0.8%), headache (0.8%), rhinitis (0.8%), tonsillitis (0.8%), tonsillitis streptococcal (0.8%), and vulvovaginitis (0.8%).

Table 3 summarizes the HCRU associated with varicella by patient group. Among outpatients, 86.7% visited the doctor’s office at least once, while 6.7% visited more than once, and 20.0% visited a hospital outpatient clinic, where 1.3% of patients visited the clinic more than once. Medication use among outpatients consisted of 96.0% using OTCs with an average of 1.9 (95% CI: 1.6, 2.3) per patient, 9.3% using prescription medications with an average of 1.1 (95% CI: 0.5, 2.1) per patient, and tests/procedures were used by 4.0% of patients. Allied health professionals were consulted by 2.7% of outpatients, for an average of 2.5 (95% CI: 0.9, 5.4) times.

**Table 3:** Varicella associated healthcare resource utilization

Type of HCRU	Outpatients (*N* = 75)		Inpatients (*N* = 81)	
	% Patients	Mean (95% CI)^a^	% Patients	Mean (95% CI)^a^
Visits to doctor’s office	86.7%	1.1 (0.9, 1.4)	44.4%	1.2 (0.9, 1.6)
Visits to ER	0.0%	N/C	2.5%	1.0 (N/C)
Visits to hospital outpatient clinic	20.0%	1.1 (0.6, 1.7)	7.4%	1.2 (0.5, 2.3)
Total outpatient visits^b^	100%	1.1 (1.0, 1.1)	48.1%	1.1 (1.0, 1.2)
Hospitalization	N/A	N/A	100%	3.6 (3.2, 4.1)
ICU stay	N/A	N/A	2.5%	6.0 (N/C)
Prescription medications	9.3%	1.1 (0.5, 2.1)	70.4%	2.0 (1.7, 2.4)
OTC medications	96.0%	1.9 (1.6, 2.3)	53.1%	2.5 (2.0, 3.0)
Tests/procedures	4.0%	1.0 (N/C)	97.5%	3.8 (3.4, 4.2)
Allied health professional consultations	2.7%	2.5 (0.9, 5.4)	30.9%	1.6 (1.1, 2.1)

*CI* confidence interval, *ER* emergency room, *HCRU* healthcare resource utilization, *ICU* intensive care unit, *N/A* not applicable, *N/C* not calculable, *OTC* over the counter

^a^Denotes the average number of times each healthcare resource was used among users; for hospitalization and hospital ICU stay, it denotes the duration of days

^b^Sum of visits to doctor’s office, ER, and hospital outpatient clinic

Among inpatients, the mean duration of hospital stay was 3.6 (95% CI: 3.2, 4.1) days. The most common resource used by inpatients was tests/procedures [97.5% of patients; mean number per patient: 3.8 (95% CI: 3.4, 4.2]. Prescription medications were recorded for 70.4% of inpatients [mean number of medications used per patient: 2.0 (95% CI: 1.7, 2.4)] and OTC medications for 53.1% [2.5 (95% CI: 2.0, 3.0) per patient]. In addition to their hospitalization, 44.4% of inpatients also visited a doctor’s office [1.2 (95% CI: 0.9, 1.6) visits per patient], 30.9% consulted an allied health professional [1.6 (95% CI: 1.1, 2.1) per patient], and 7.4% visited a hospital outpatient clinic [1.2 (95% CI: 0.5, 2.3) per patient]. Finally, an average of one visit to an ER was reported for 2.5% of inpatients and 2.5% spent time in the ICU with a mean number of 6 days per patient.

Table 4 provides the direct and indirect associated costs per varicella case for outpatients and inpatients by type of resource utilized as a result of varicella infection. The overall mean direct cost per patient for outpatients in this study was 16,174.6 (95% CI: 13,500.0, 18,849.0) HUF and for inpatients was 171,739.7 (95% CI: 151,691.0, 191,789.0) HUF. For outpatients, prescription medication cost accounted for most of the overall direct cost [mean (95% CI): 9733.1 (9650.9, 9815.3) HUF], whereas hospitalization cost [mean (95% CI): 123,889.0 (109,453.0, 138,325.0) HUF] made up the predominant portion of the overall direct costs for inpatients. Tests/procedures were the resources associated with the second highest cost [mean (95% CI): 13,648.0 (11,645.0, 15,651.0) HUF] for inpatients. The indirect cost of varicella was a considerable amount for both outpatients and inpatients, with mean costs of 33,616 HUF and 56,407.0 HUF per case, respectively.

**Table 4:** Cost (HUF / €) per pediatric case of varicella

	Outpatients (*N* = 75)			Inpatients (*N* = 81)		
	Mean Cost^a^		95% CI	Mean Cost^a^		95% CI
Direct costs						
Visits to doctor’s office	HUF €	2880.0 9.3	(2513.4, 3246.6) (8.1, 10.5)	HUF €	1592.6 5.1	(1159.0, 2026.1) (3.7, 6.5)
Visits to ER	HUF €	0.0	N/C	HUF €	555.6 1.8	(0.0, 1332.4) (0.0, 4.3)
Visits to hospital outpatient clinic	HUF €	2133.3 6.9	(1111.8, 3154.8) (3.6, 10.2)	HUF €	864.2 2.8	(147.9, 1580.5) (0.5, 5.1)
Hospitalization	HUF €	N/A	N/A	HUF €	123,889.0 399.7	(109,453.0, 138,325.0) (353.1, 446.2)
ICU stay	HUF €	N/A	N/A	HUF €	11,111.0 35.8	(0.0, 26,648.0) (0.0, 86.0)
Prescription medications	HUF €	9733.1 31.4	(9650.9, 9815.3) (31.1, 31.7)	HUF €	11,370.0 36.7	(10,895.0, 11,845.0) (35.1, 38.2)
OTC medications	HUF €	109.5 0.4	(89.9, 129.1) (0.3, 0.4)	HUF €	42.6 0.1	(20.3, 64.9) (0.1, 0.2)
Tests/procedures	HUF €	118.7 0.4	(0.0, 277.3) (0.0, 0.9)	HUF €	13,648.0 44.0	(11,645.0, 15,651.0) (37.6, 50.5)
Allied health professional consultations	HUF €	1200.0 3.9	(0.0, 3165.4) (0.0, 10.2)	HUF €	8666.7 28.0	(5389.9, 11,943.0) (17.4, 38.5)
Overall direct costs	HUF €	16,174.6 52.2	(13,500.0, 18,849.0) (43.5, 60.8)	HUF €	171,739.7 554.0	(151,691.0, 191,789.0) (489.3, 618.7)
Indirect costs						
Lost work by caregivers	HUF €	33,616.0 108.4	N/C	HUF €	56,407.0 182.0	(50,008.0, 62,806.0) (161.3, 202.6)
Total	HUF €	49,790.6 160.6	(47,116.5, 52,465.0) (152.0, 169.2)	HUF €	228,146.7 736.0	(203,269.5, 253,025.1) (655.7, 816.2)

*CI* confidence interval, *ER* emergency room, *HUF* Hungarian Forint, *ICU* intensive care unit, *N/A* not applicable, *N/C* not calculable, *OTC* over the counter

^a^Mean (95% CI) among all patients. Based on patients with available information

Table 5 presents the estimated annual costs (direct, indirect and total) associated with varicella among children <15 years of age in Hungary. These estimates were based on the cost per varicella case reported in Table 4, the number of varicella cases reported for 2015 in Hungary (*n* = 40,853), the proportion of varicella cases in Europe attributed to patients <15 years of age (92%) and the hospitalization rate in children with varicella in this age group reported for 2010 (0.48%) [9, 10]. Based on an estimated annual incidence of 37,585 pediatric (< 15 years of age) varicella cases, consisting of 179 inpatients and 37,406 outpatients, the total estimated annual direct and indirect costs associated with varicella in pediatric patients in Hungary for 2015 are 635,798,607.6 HUF (€ 2,036,465.25) and 1,267,533,916.7 HUF (€ 4,059,946.44), respectively, for a total cost of 1,903,332,524.3 HUF (€ 6,096,449). A sensitivity analysis of the extrapolated costs was also performed (Appendix: Tables 6 and 7) due to the range of values for the cost of doctor’s office and ER visits, hospitalization, and ICU stay (Table 1), where the lowest (Scenario 2; Appendix: Table 6) and highest (Scenario 3; Appendix: Table 7) values were taken into consideration. The results of this analysis indicated that the total annual cost for varicella in Hungary could be between 1,823,225,470.8 HUF (€ 5,881,563.6) and 1,983,439,572.5 HUF (€ 6,398,400.1).

**Table 5:** Estimated annual (2015) costs (HUF / €) for children with varicella in Hungary^a^

	Annual Cost (HUF / €)		(%) of Total Direct Cost
Direct costs			
Visits to doctor’s office	HUF €	108,013,379.8 348,441.5	17.0%
Visits to ER	HUF €	99,568.0 321.2	0.0%
Visits to outpatient clinic	HUF €	79,952,119.2 257,918.4	12.6%
Hospitalization	HUF €	22,203,503.7 71,626.5	3.5%
ICU stay	HUF €	1,991,323.9 6423.8	0.3%
Prescription medications	HUF €	366,109,594.3 1,181,036.8	57.6%
OTC medications	HUF €	4,104,294.8 13,240.1	0.6%
Tests/procedures	HUF €	6,884,922.8 22,210.1	1.1%
Allied health professional consultations	HUF €	46,439,901.0 149,811.0	7.3%
Total direct costs	HUF €	635,798,607.6 2,051,029.4	N/A
Indirect costs			
Lost work by caregivers	HUF €	1,267,533,916.7 4,088,951.0	N/A
Total	HUF €	1,903,332,524.3 6,139,980.4	N/A

*ER* emergency room, *HUF* Hungarian Forint, *ICU* intensive care unit, *N/A* not applicable, *OTC* over the counter

^a^Annual number of cases (*n* = 37,585) are estimated pediatric cases (< 15 years old) for 2015 based on the European varicella surveillance report from 2010 [9], and the National Centre for Epidemiology’s report on the 2015 epidemiological situation in Hungary [10]

## Discussion

This study was conducted to evaluate the HCRU and associated costs attributable to varicella in pediatric patients seen either as outpatients or as inpatients in Hungary, as well as to describe the severity of disease and types of complications exhibited by children with varicella in Hungary. We demonstrated that substantial clinical and healthcare burden is associated with varicella in both the outpatient and inpatient pediatric population in Hungary.

Varicella-related complications were more common in inpatients than in outpatients with varicella (92.6% vs 12.0%, respectively). These results are consistent with previously published data from Italy, Germany, and Switzerland. Complication rates of 3.5%, 5.9% and 12.0% were reported for Italy, Germany, and Switzerland, respectively, among pediatric patients presenting with varicella in an outpatient setting [30–32]. However, it is important to note that the study in Switzerland included outpatients only, while some of the patients who experienced complications in the Italian and German studies were later hospitalized. The rates of varicella-related complications reported for inpatient pediatric cases in Germany, Turkey, and Belgium were 65.0%, 79.0%, and 79.6%, respectively [33–35]. The most common types of complications reported in our study in Hungary included keratoconjunctivitis, dehydration, skin and soft tissue infection, bronchitis, pneumonia, facial paresis, severe pain and cerebellitis. These findings are consistent with the commonly reported complications in other studies conducted in European countries [36–44]. No deaths were reported in our study; however, this was expected due to the low sample size and the varicella case-fatality rate which has been estimated at 1 death per 100,000 children [45–47].

Among the study population, 44.4% of inpatients and 86.7% of outpatients had visited a doctor’s office at least once for varicella during their illness, with average visits per patient of 1.2 and 1.1 times, respectively. Among inpatients a mean of 3.6 days of hospital stay was reported in our study, which is in line with the average of three to eight days previously reported in other European studies [34, 46, 48–50].

In Hungary, varicella vaccination is presently optional, with no specific recommendation in the national immunization plan. In Germany, where varicella vaccination is universally recommended at a national level for children 11–14 months of age, with a second dose at 15–23 months, a recent study (2013) reported a decrease in the number of cases of children with varicella by 67% during a five-year observation period after recommending the vaccine [51]. Furthermore, varicella complications were rarely observed in Germany after the vaccine recommendation was implemented, reported in less than 1% of cases, and pediatric hospitalizations decreased by 43% during the observation period. Germany’s vaccination program demonstrated success despite its suboptimal vaccination coverage, indicating the importance of implementing a national vaccination program in countries lacking one.

Tóth et al. previously estimated the total indirect cost for varicella in children under the age of 15 (*n* = 38,316) in Hungary for 2010 at € 1,362,897, equivalent to 375,409,978.7 HUF [52]. Here, we report a total estimated annual indirect cost of 1,267,533,916.7 HUF for 37,585 pediatric cases. The ~3 fold higher cost found in our study is primarily attributable to the daily salary rate utilized of 13,313 HUF/day [based on data obtained from the OECD] vs the daily salary of 3100 HUF/day assumed by the prior study. The Tóth et al. study only presented costs for inpatient and outpatient care and medication use combined for all ages, and their estimates were based only on data that was recorded through the national health insurance funds. The cost estimates for hospital inpatient care were actually lower in the present study (77,519,651.40 HUF vs 90,445,038.30 HUF), which is not surprising given that the Tóth et al. study included all age groups, and that the full costs of hospitalization are covered by the national health insurance system in Hungary. For both outpatient care and medications, however, the estimates differ substantially (188,065,067.00 HUF vs 1,620,307.10 HUF for outpatient care, and 48,761,922.48 HUF vs 370,213,889.10 HUF for medications). The difference observed for outpatient care in the two estimates may be attributable to the inclusion of costs related to seeking care outside the reimbursed system in Hungary, and that in our analysis, we have assumed that all patients would seek care, which may have led to an overestimation of outpatient care. For medications, the difference is likely attributable to several factors; the inclusion of OTC, non-reimbursed medications and co-payments in the present study vs the payer perspective in the prior study, increases in the cost of medications, and our assumption that all varicella patients would be treated with medications.

Our cost estimates reflect those reported for other European countries, including Italy, Spain and Germany. In Italy, the cost per child (1–14 years) with varicella, based mainly on uncomplicated cases (96.5% of all cases), was estimated at US $146.9 (equivalent to 28,004.3 HUF; cost in 1997 HUF [53]), as compared to the average of 33,616 HUF (€ 107.68) per outpatient case estimated in our study [30]. In Spain, the total cost per child (≤ 14 years) with varicella was estimated at € 108.67 [54], equivalent to 27,354.4 HUF (cost in 2004 HUF), while Germany reported an estimated cost of € 162.5 [55] per child (≤ 12 years), equivalent to 39,484.3 HUF (cost in 2002 HUF). Average healthcare expenditure per capita in 2014 was $5411 USD / € 4058 in Germany, $2658 USD / € 1994 in Spain, and $1037 USD / € 778 in Hungary) [56], indicating that the relative cost of treating varicella, as compared to per capita health expenditure, is considerably higher in Hungary than in other European countries.

European countries which have implemented routine varicella vaccination programs, such as Germany, have shown substantial reductions (77%) in disease incidence [51], supporting the view that routine vaccination could reduce the economic burden of varicella in Hungary. Currently, private sector vaccination is offered in Hungary at a cost per dose of 21 USD, or 5858.6 HUF, with a recommended two dose schedule concurrent with MMR vaccines administered at 15 months and 11 years, or else at 15 months and 18 months. The estimated cost of a single dose programme to vaccinate the entire birth cohort of Hungary (*n* = 91,700) would be 537,233,620.0 HUF, or € 1,729,892.3, and would likely reduce the varicella incidence by 52% in the first year and by 99% after 10 years. Compared to the cost of treatment of varicella, vaccination is highly likely to be a cost-effective intervention in Hungary [14].

The limitations of this study include the retrospective chart review design, as only a cross-section of patient care may have been captured, which could have resulted in an underestimation of the associated HCRU. Furthermore, the possible selection bias of cases seeking help may have exaggerated the estimation of the burden associated with varicella. No assessment of out of pocket patient expenses was made, and only a limited assessment of indirect cost due to work loss was assessed. Finally, the relatively small sample size of the study and the small number of participating sites may reduce the external validity of the findings which may not be fully representative of the routine care of varicella in all clinical settings.

## Conclusion

Varicella is associated with substantial clinical burden in Hungary resulting in the utilization of a significant amount of healthcare resources and considerable economic burden. Despite the fact that the estimated cost of treating each varicella case in Hungary seems to be comparable to other European countries, such as Spain and Germany, the relative cost to per capita healthcare expenditure is considerable higher in Hungary. These results support the need for routine childhood varicella vaccination to reduce the varicella-associated disease burden in Hungary.

## Appendix

**Table 6:** Scenario 2^a^: Estimated annual (2015) costs (HUF / €) for children with varicella in Hungary

	Annual Cost (HUF / €)		(%) of Total Direct Cost
Direct costs			
Visits to doctor’s office	HUF €	36,004,458.7 116,147.2	6.5%
Visits to ER	HUF €	66,378.1214.1	0.0%
Visits to outpatient clinic	HUF €	79,952,119.2 257,918.4	14.4%
Hospitalization	HUF €	14,802,322.1 47,751.0	2.7%
ICU stay	HUF €	1,327,563.0 4282.6	0.2%
Prescription medications	HUF €	366,109,594.3 1,181,036.8	65.9%
OTC medications	HUF €	4,104,294.8 13,240.1	0.7%
Tests/procedures	HUF €	6,884,922.8 22,210.1	1.2%
Allied health professional consultations	HUF €	46,439,901.0 149,811.0	8.4%
Total direct costs	HUF €	555,691,554.1 1,792,611.6	N/A
Indirect costs			
Lost work by caregivers	HUF €	1,267,533,916.7 4,088,951.0	N/A
Total	HUF €	1,823,225,470.8 5,881,563.6	N/A

*ER* emergency room, *HUF* Hungarian Forint, *ICU* intensive care unit, *N/A* not applicable, *OTC* over the counter

^a^ Scenario 2 = lowest cost scenario; based on the lowest value in the key unit cost range for doctor’s office and ER visits, hospitalization, and ICU stay (Table 1)

**Table 7:** Scenario 3^a^: Estimated annual (2015) costs (HUF / €) for children with varicella in Hungary

	Annual Cost (HUF / €)		(%) of Total Direct Cost
Direct costs			
Visits to doctor’s office	HUF €	180,022,297.2 580,736.0	25.1%
Visits to ER	HUF €	132,756.1 428.3	0.0%
Visits to outpatient clinic	HUF €	79,952,119.2 257,918.4	11.2%
Hospitalization	HUF €	29,604,646.0 95,502.0	4.1%
ICU stay	HUF €	2,655,124.3 8565.2	0.4%
Prescription medications	HUF €	366,109,594.3 1,181,036.8	51.1%
OTC medications	HUF €	4,104,294.8 13,240.1	0.6%
Tests/procedures	HUF €	6,884,922.8 22,210.1	1.0%
Allied health professional consultations	HUF €	46,439,901.0 149,811.0	6.5%
Total direct costs	HUF €	715,905,655.7 2,309,448.1	N/A
Indirect costs			
Lost work by caregivers	HUF €	1,267,533,916.7 4,088,951.0	N/A
Total	HUF €	1,983,439,572.5 6,398,400.1	N/A

*ER* emergency room, *HUF* Hungarian Forint, *ICU* intensive care unit, *N/A* not applicable, *OTC* over the counter

^a^Scenario 3 = highest cost scenario; based on the highest value in the key unit cost range for doctor’s office and ER visits, hospitalization, and ICU stay (Table 1)

## Abbreviations

ACV: Acyclovir
CI: Confidence interval
ER: Emergency room
GCP: Good clinical practices
HCRU: Healthcare resource utilization
HUF: Hungarian forint
ICU: Intensive care unit
IEC: Independent ethics committee
MedDRA: Medical dictionary for regulatory activities
MISEK: Miskolci Semmelweis Hospital and University Teaching Hospital
MOH: Ministry of health
NCE: National center for epidemiology
NIP: National immunization program
OECD: Organization for economic co-operation and development
OTC: Over the counter
SD: Standard deviation
USD: United States Dollar
VZV: Varicella zoster virus
